# Whole-Genome Resequencing of Holstein Bulls for Indel Discovery and Identification of Genes Associated with Milk Composition Traits in Dairy Cattle

**DOI:** 10.1371/journal.pone.0168946

**Published:** 2016-12-28

**Authors:** Jianping Jiang, Yahui Gao, Yali Hou, Wenhui Li, Shengli Zhang, Qin Zhang, Dongxiao Sun

**Affiliations:** 1 Department of Animal Genetics and Breeding, College of Animal Science and Technology, Key Laboratory of Animal Genetics and Breeding of the Ministry of Agriculture, National Engineering Laboratory for Animal Breeding, China Agricultural University, Beijing, China; 2 Laboratory of Disease Genomics and Individualized Medicine, Beijing Institute of Genomics, Chinese Academy of Sciences, Beijing, China; Midwestern University, UNITED STATES

## Abstract

The use of whole-genome resequencing to obtain more information on genetic variation could produce a range of benefits for the dairy cattle industry, especially with regard to increasing milk production and improving milk composition. In this study, we sequenced the genomes of eight Holstein bulls from four half- or full-sib families, with high and low estimated breeding values (EBVs) of milk protein percentage and fat percentage at an average effective depth of 10×, using Illumina sequencing. Over 0.9 million nonredundant short insertions and deletions (indels) [1–49 base pairs (bp)] were obtained. Among them, 3,625 indels that were polymorphic between the high and low groups of bulls were revealed and subjected to further analysis. The vast majority (76.67%) of these indels were novel. Follow-up validation assays confirmed that most (70%) of the randomly selected indels represented true variations. The indels that were polymorphic between the two groups were annotated based on the cattle genome sequence assembly (UMD3.1.69); as a result, nearly 1,137 of them were found to be located within 767 annotated genes, only 5 (0.138%) of which were located in exons. Then, by integrated analysis of the 767 genes with known quantitative trait loci (QTL); significant single-nucleotide polymorphisms (SNPs) previously identified by genome-wide association studies (GWASs) to be associated with bovine milk protein and fat traits; and the well-known pathways involved in protein, fat synthesis, and metabolism, we identified a total of 11 promising candidate genes potentially affecting milk composition traits. These were *FCGR2B*, *CENPE*, *RETSAT*, *ACSBG2*, *NFKB2*, *TBC1D1*, *NLK*, *MAP3K1*, *SLC30A2*, *ANGPT1* and *UGDH*. Our findings provide a basis for further study and reveal key genes for milk composition traits in dairy cattle.

## Introduction

In recent years, the establishment of next-generation sequencing (NGS) technology has led to substantial progress in terms of the discovery of large numbers of single-nucleotide polymorphisms (SNPs), insertions and deletions (indels), and large structural variations (SVs). Short indels are now recognized as the second most common form of genomic variation [1], which contribute to phenotypic diversity in many species and human diseases [2], such as cystic fibrosis [3] and fragile X syndrome [4]. In cattle, indels have been shown to contribute to complex traits, such as the double-muscled phenotype [5]. Since Mills et al. constructed the initial map of human indel variations [6], and with the increased availability and advances of high-throughput sequencing technology, indels have been discovered in individual genomes across different species [1, 7–12]. At the time of writing, more than 8 million indels are registered in the dbSNP bovine database (ftp://ftp.ncbi.nih.gov/snp/organisms/cow_9913/VCF/, updated in July 11, 2016). Therefore, it is now feasible to identify and characterize the molecular basis of complex traits using indels.

Milk production in dairy cattle is an economically important variable, especially the traits of milk yield and milk composition. Numerous studies have focused on the detection of quantitative trait loci (QTL) for milk production traits [13–19], since the first report on QTL mapping in dairy cattle by Georges et al. [20]. In recent decades, based on QTL mapping and genome-wide association studies (GWASs), numerous candidate genes and mutations associated with milk production traits have been identified, including two confirmed causal mutations: *DGAT1* p.Lys232Ala and *GHR* p.Phe279Tyr, which have large effects on milk yield and composition [21, 22]. However, to date, only limited investigations to identify genes associated with milk production traits using whole-genome resequencing have been reported [23–25].

The aims of this study were thus to detect indels across the whole genome in eight Holstein bulls with extremely high and low estimated breeding values (EBVs) of milk protein percentage and fat percentage using NGS, and to explore the short indels [1–49 base pairs (bp)] that were polymorphic between these two groups and to identify functional genes that are important for milk protein and fat traits.

## Materials and Methods

Frozen semen samples were collected along with the regular quarantine inspection of the farms and in strict accordance with the protocol approved by the Institutional Animal Care and Use Committee (IACUC) at China Agricultural University.

### Sample collection

Eight bulls from two half-sib families and two full-sib families (n = 2 each) were selected from the Beijing Dairy Cattle Center (http://www.bdcc.com.cn/), according to their EBVs of milk protein percentage (PP) and fat percentage (FP), which were calculated based on a multiple trait random regression test-day model using the software RUNGE by the Dairy Data Center of China (http://www.holstein.org.cn/). The reliability of EBVs for milk protein percentage and fat percentage of each bull was more than 0.80. The two bulls in each group showed high and low EBVs for milk protein percentage and fat percentage. Detailed information on the eight bulls is presented in Table 1.

**Table 1:** Descriptive statistics of PP and FP EBVs for eight bulls.

Sib-family	Sample	EBV for PP	EBV for FP	Reliability for PP	Reliability for FP
Full-sib1	1	0.03	0.10	0.99	0.99
	2	-0.13	-0.31	0.97	0.87
Full-sib2	3	0.08	0.56	0.99	0.98
	4	-0.03	0.27	0.98	0.98
Half-sib1	5	0.22	0.09	0.91	0.8
	6	0.01	-0.26	0.99	0.99
Half-sib2	7	0.07	-0.14	0.98	0.99
	8	-0.06	-0.26	0.99	0.99

### DNA library construction and sequencing

Genomic DNA was extracted from frozen semen samples using the standard phenol/chloroform extraction method. DNA degradation and contamination were monitored on 1% agarose gels and the concentration and purity were assessed on NanoDrop 2000 (Thermo Scientific Inc. Waltham, DE, USA); the high-quality DNAs were then used for library construction. Two paired-end libraries were constructed for each individual, the read length was 2×100 bp, and sequencing was performed using Illumina Hiseq2000 instruments (Illumina Inc., San Diego, CA, USA).

### Read mapping and variant calling

The cattle reference genome assembly (UMD3.1.69) was downloaded from the Ensembl Genome Browser website (http://asia.ensembl.org). We applied the NGS QC Toolkit [26] with default parameters to minimize mapping errors. To map reads to the reference genome, we used the Burrows–Wheeler Alignment tool (BWA ver. 0.6.2) [27], mainly with the default parameters. To call indels, SAMtools (ver. 0.1.19) [28] was applied. The criteria were as follows: overall base quality score≥20; read depth <100 for each individual; and alternative allele on either forward or reverse supporting reads >3.

### Indel validation

To evaluate the reliability of the resequencing data, 20 randomly selected indels were validated using a PCR assay and direct sequencing. PCR primers were designed using PRIMER3 (http://frodo.wi.mit.edu/primer3) and Oligo 6.0 to amplify the 150–300 bp fragments containing the indels (S1 Table). The PCR reaction was performed in a volume of 25 μl containing 50–100 ng of genomic DNA, 10 μl of premix, 1 μl of each primer, and 11 μl of PCR buffer, using the following PCR program: 94°C for 10 min; 35 cycles of 94°C for 40 s, 55–60°C for 40 s, and 72°C for 40 s; and then 72°C for 7 min at the end of the final cycle. The PCR products were purified and then subjected to Sanger sequencing.

### Detection of differential variants and functional annotation

Among all of the indels from the eight bulls retained after filtering, we selected those that were polymorphic between the two groups with high and low EBVs, which were called the ‘common differential variants’. The pipeline in this study was as follows: we first collected all the variants that were polymorphic between the two individuals within each family, and then retained the common differential variants with the same allelic distribution directions between the high and low individuals across the four families. Subsequently, these common differential indels were annotated by ANNOVAR [29] based on the cattle gene set downloaded from the Ensembl website (ftp://ftp.ensembl.org/pub/release-79/gtf/bos_taurus) to identify the indels within functional genes. Moreover, we compared the differential indels mentioned above with the variants in cattle SNP database (http://www.ncbi.nlm.nih.gov).

### Integrated analysis of resequencing, QTL, and GWAS data and known pathways

We specifically focused on those common differential indels between the high and low groups located in genic regions or flanking regions of genes to identify candidate genes and causal mutations related to milk composition traits. On the one hand, we estimated the positions of genes containing these indels using QTL mapper v.2.019 (www.animalgenome.org/cgi-bin/QTLdb/), and then compared the genetic positions of the genes with the confidence intervals and peak positions of previously reported QTL (http://www.animalgenome.org/cgi-bin/QTLdb, updated in April 29, 2016). We also compared the physical chromosomal positions of the above genes with the SNPs determined by previous GWASs [13, 15] to be significantly associated with milk protein and fat traits of dairy cattle. Then, we performed Gene Ontology (GO) functional annotation and Kyoto Encyclopedia of Genes and Genomes (KEGG) pathway analysis of the genes containing the identified indels using KOBAS tool [30]. During the functional annotation of genes, a *P* value of <0.05 determined by Fisher’s exact test was set as the criteria for significance. In addition, we downloaded the genes involved in well-known pathways for protein, fat, and fatty acid metabolism from the KEGG pathway website (http://www.kegg.jp/), consisting the mTOR, insulin, AMPK, PPAR, Jak-STAT, PI3K-Akt, MAPK, and TGF-β signaling pathways, and further determined whether these genes were included in the gene list which were identified by integrated analysis of QTL and GWAS data.

## Results

### Data production and read mapping

After resequencing of the genomic DNA samples for eight Holstein bulls, a total of 2,303,781,448 short reads were obtained, and the average proportion of uniquely mapped reads was as high as 82.62%. The sequencing depth ranged from 7× to 10×, with an average of 8.1×, and the genome coverage was approximately 98% in each individual (Table 2).

**Table 2:** Summary of sequencing, mapping statistics, and indel count for individuals.

Sample	Raw reads	Mapped reads	Uniquely mapped reads (%)	Genome coverage (%)	Sequencing depth (X)	Indel
1	289,952,310	261,783,075	83.75	98.58	8	363,757
2	286,870,238	252,201,294	81.15	98.55	8	346,453
3	292,878,886	257,840,281	81.69	98.59	8	366,402
4	272,948,496	241,748,531	82.97	98.52	8	360,827
5	251,953,446	218,677,291	82.90	98.34	7	323,490
6	337,815,303	314,651,325	80.88	98.40	10	411,070
7	288,003,254	253,311,627	82.45	98.61	8	368,232
8	283,359,516	255,124,411	85.16	98.57	8	381,119
Average	287,972,681	256,917,229	82.62	98.52	8.1	365,169

### Indel detection

**Fig 1:**
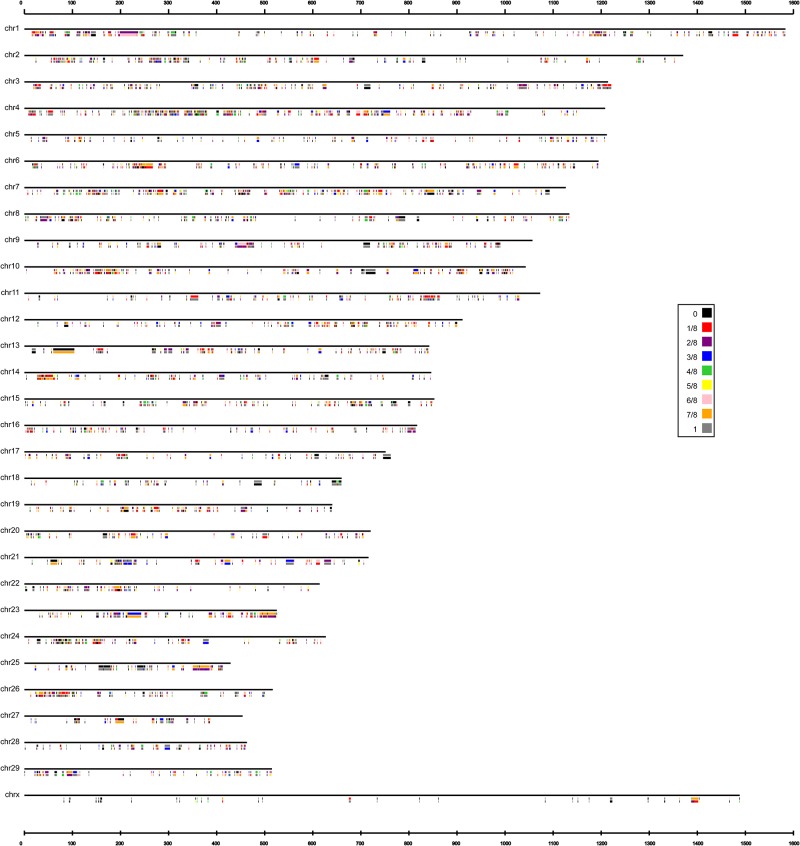
Distribution of allele frequencies for the common differential indels between high and low groups. The first line represents insertion frequency for each common differential indel locus in low group, and the second line represents insertion frequency for each common differential indel locus in high group.

In total, 912,302 nonredundant short indels (1–49 bp) were obtained from the eight bulls after filtering using SAMtools. The number of indels detected in each bull varied from 323,490 to 411,070, with an average of 365,169 (Table 2). Among them, we focused on the unique indels that were polymorphic between the bulls with high and low EBVs of milk protein percentage and fat percentage, which were inferred to be relevant to the milk protein and fat trait. As a result, after filtering, we obtained 3,625 common differential indels that were polymorphic between the high and low groups of bulls. The distribution of allele frequencies of these common differential indels was calculated and presented in Fig 1. We further compared our results with the variants in the SNP database (NCBI dbSNP, updated in July 11, 2016) and found that the majority of the common differential indels (76.67%) were novel.

To estimate the accuracy and reliability of our resequencing data, PCR amplification and Sanger sequencing were applied to validate 20 randomly selected indels with a range of lengths from 1 to 21 bp with the same genomic DNA samples of the eight bulls as used for resequencing. We found that the sequences of the 14 indels were consistent with those from our whole-genome resequencing data, indicating that the accuracy of our results was 70% (S1 Table). The primer sequences are shown in the supplemental material (S1 Table).

### Functional annotation and genomic distribution

**Fig 2:**
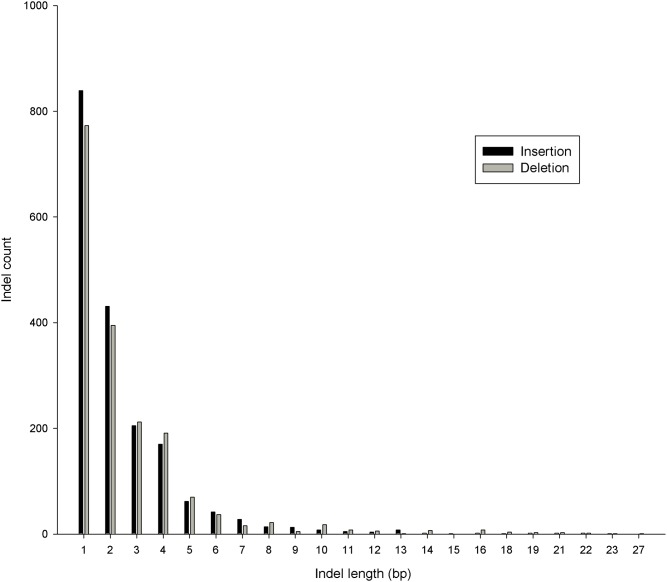
Distribution of indel length (bp). The indel represents the common differential one between the high and low groups.

**Fig 3:**
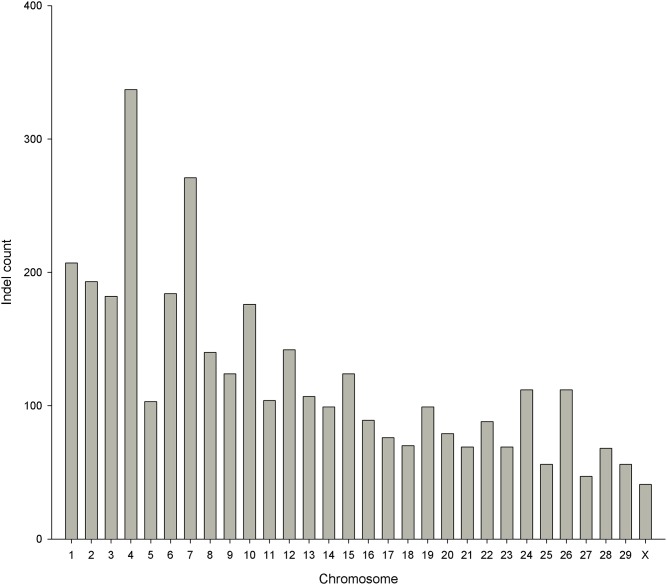
The number of indels in each chromosome. The indel represents the common differential one between the high and low groups.

Among the 3,625 common indels that were polymorphic between the high and low bull groups, the largest indel was 27 bp, but most (97.96%) of them were less than 10 bp. Single-bp indels (44.47%) were the most common, and the number of insertions (n = 1,842) was slightly more than the number of deletions (n = 1,783). The cumulative length of the indels reached 9 kb. The distribution of indel length is shown in Fig 2. The indels were proportionally distributed among autosomes corresponding to chromosome length; the number of indels was lower on X chromosome than on autosomes (Fig 3).

Functional annotation of the 3,625 common differential indels was performed against known genes from the Ensembl website using ANNOVAR software (Table 3). As a result of this, the majority (68.634%) of indels was found to be located in intergenic regions, and the remaining 1,137 indels were within 767 genes, including in introns (29.379%), exons (0.138%), 3’ untranslated region (3’ UTR) (0.276%), and other regions (ncRNAs, splicing site, etc.). Regarding the exonic indels that were identified, there were 2 frameshift indels with lengths not divisible by 3, and 3 nonframeshift indels, called 3n indels [31], with lengths divisible by 3.

**Table 3:** Annotation of 3,625 common differential indels for eight bulls.

Category	Indel	Indel %^1^
All	3625	100
Intergenic	2488	68.634
Upstream	30	0.828
Downstream	24	0.662
Upstream;downstream^a^	1	0.028
3’ UTR	10	0.276
Splicing	1	0.028
ncRNA_exonic	1	0.028
Intronic	1065	29.379
Exonic		0.138
Non-frameshift deletion	1	
Non-frameshift insertion	2	
Frameshift deletion	1	
Frameshift insertion	1	

^1^Percentage was calculated based on total annotated indels.

^a^Variant located in both upstream and downstream regions (possibly for two different genes).

### Integrated analysis with known QTL and significant SNPs identified by GWASs

In this part of the study, we first compared the genetic positions of the 1,137 indels located in 767 genes with QTL known to be associated with milk protein and fat traits in the Cattle QTL database (http://www.animalgenome.org/cgi-bin/QTLdb/BT/index). We found that 869 indels, corresponding to 605 unique genes, overlapped with the confidence intervals of 538 known QTL. Notably, 542 of them were located in 372 functional genes that were close to the peak position of the known QTL with a genetic distance of less than 5 cM. Furthermore, of the 1,137 indels, 926 located in 635 genes were close (< 5 Mb) to significant SNPs identified by previous GWASs. Hence, a total of 695 of the genes in these two groups were retained for further analyses.

### Gene Ontology and pathway analyses

We performed GO enrichment and KEGG pathway analyses using the KOBAS tool based on the 695 genes mentioned above. In total, 306 GO terms and 29 KEGG pathways were significantly enriched (*P*<0.05; S2 Table), mainly including ‘inorganic cation transmembrane transport’, metabolic process related to protein and lipid metabolism such as ‘lipid biosynthetic process’, ‘phosphatidylglycerol biosynthetic process’, ‘glycosaminoglycan biosynthetic process’, ‘protein kinase A binding’, ‘protein kinase C activity’ and ‘protein phosphatase inhibitor activity’ *etc*., and terms associated with the cell cycle and cell adhesion such as ‘regulation of cell cycle G_1_/S phase transition’ and ‘protein complex involved in cell adhesion’. In addition, significant enriched KEGG pathways consisted of ‘thyroid hormone synthesis’, ‘aldosterone synthesis and secretion’, ‘GnRH signaling pathway’, MAPK signaling pathway, ‘gap junction’, and ‘phosphatidylinositol signaling system’ *et al*.

We also found that 34 out of the 695 genes with an indel polymorphic between the high and low groups of bulls were involved in several well-known pathways for protein and fat synthesis and metabolism. These included the mTOR and Jak-STAT signaling pathways, both of which play pivotal roles in the synthesis of milk proteins; the AMPK signaling pathway as an energy supplement system; the insulin and PPAR signaling pathway for fat synthesis; the PI3K-Akt signaling pathway together with the MAPK signaling pathway, which play roles in cellular functions, such as transcription, translation, and cell proliferation; and the TGF-β signaling pathway, which is essential for mammary epithelial cell proliferation.

### Identification of candidate genes associated with milk protein and fat traits

Based on the known QTL and significant SNPs identified by previous GWASs, along with biological functions of genes, 11 genes that containing at least one common differential indel locus were considered as promising candidates for an association with milk protein and fat traits. Of them, four genes, including Acyl-CoA synthetase bubblegum family member 2 (*ACSBG2*), nuclear factor kappa B subunit 2 (*NFKB2*), TBC1 domain family member 1 (*TBC1D1*) and mitogen-activated protein kinase kinase kinase 1 (*MAP3K1*), were located near to both the peak locations of the reported QTL and significant SNPs for milk protein and fat traits, and were involved in the well-known protein and lipid metabolic pathways. The Fc fragment of IgG receptor IIb (*FCGR2B*) and centromere protein E (*CENPE)* genes were located in QTL regions and near to the significant SNPs reported to be associated with milk protein and fat traits. Two genes, solute carrier family 30 member 2 (*SLC30A2*) and UDP-glucose 6-dehydrogenase (*UGDH*) were very close to the peak position of QTL and significant SNPs for milk protein and fat traits. Two genes, nemo like kinase (*NLK*) and angiopoietin 1 (*ANGPT1*), were near to the significant SNPs for milk protein percentage, and participated in the MAPK and PI3K-Akt signaling pathways. In addition, retinol saturase (*RETSAT*) was also near to significant SNPs for milk protein and fat traits. The chromosomal positions of these 11 genes and detailed information about their nearest QTL and significant SNPs were shown in Tables A and B in S1 File. The indel genotypes in these 11 genes between high and low groups were shown in Table 4.

**Table 4:** The indel genotypes in 11 genes between high and low groups.

Gene name	Indel	Location	Indel sequence	Indel genotypes in high group				Indel genotypes in low group			
				sample1	sample3	sample5	sample7	sample2	sample4	sample6	sample8
*FCGR2B*	1N ins	exon	G	ins/del	del/del	ins/del	del/del	ins/ins	ins/del	ins/ins	ins/del
*CENPE*	3N ins	exon	AGA	del/del	del/del	del/del	del/del	ins/del	ins/del	ins/ins	ins/ins
	3N del	exon	TAG	ins/ins	ins/ins	del/ins	ins/ins	del/ins	del/ins	del/del	del/del
	3N ins	intron	GTT	del/del	del/del	del/del	ins/del	ins/del	ins/del	ins/ins	ins/ins
	1N ins	intron	T	del/del	del/del	ins/del	ins/del	ins/del	ins/del	ins/ins	ins/ins
	1N del	intron	A	ins/ins	ins/ins	ins/ins	del/ins	del/del	del/ins	del/del	del/del
	21N ins	intron	ACTTAAGTATATAACCTTAAC	del/del	del/del	del/del	del/del	ins/del	ins/del	ins/ins	ins/ins
	2N del	intron	CC	ins/ins	ins/ins	del/ins	del/ins	del/ins	del/ins	del/del	del/del
	1N del	intron	C	ins/ins	ins/ins	ins/ins	del/ins	del/ins	del/ins	del/del	del/del
	4N ins	intron	ACAC	ins/del	ins/ins	ins/ins	ins/ins	del/del	ins/del	del/del	del/del
*RETSAT*	2N del	3'UTR	AA	del/del	del/ins	del/del	del/ins	ins/ins	ins/ins	del/ins	ins/ins
	9N ins	intron	ATTCTGGGG	ins/ins	ins/del	ins/ins	ins/del	del/del	del/del	del/del	del/del
*ACSBG2*	3N ins	upstream	GGC	ins/ins	ins/ins	ins/ins	ins/ins	del/del	del/del	ins/del	ins/del
	1N ins	intron	C	ins/ins	ins/ins	ins/ins	ins/ins	del/del	del/del	ins/del	ins/del
*NFKB2*	2N ins	upstream	GG	ins/ins	ins/ins	ins/ins	ins/ins	del/del	del/del	del/del	del/del
*TBC1D1*	1N del	intron	T	ins/ins	ins/ins	ins/ins	ins/ins	del/ins	del/ins	del/ins	del/ins
*NLK*	1N ins	intron	T	ins/ins	ins/del	ins/ins	ins/ins	del/del	del/del	ins/del	ins/del
	2N del	intron	AT	del/ins	del/ins	del/del	del/del	ins/ins	ins/ins	del/ins	del/ins
	1N del	intron	A	del/ins	del/ins	del/del	del/del	ins/ins	ins/ins	del/ins	del/ins
	4N del	intron	AAAA	ins/ins	del/ins	ins/ins	ins/ins	del/ins	del/del	del/ins	del/del
*MAP3K1*	5N del	intron	CATTT	ins/ins	ins/ins	ins/ins	del/ins	del/del	del/del	del/ins	del/del
*SLC30A2*	6N del	intron	TTTTTG	del/ins	del/ins	del/ins	del/ins	ins/ins	ins/ins	ins/ins	ins/ins
*ANGPT1*	2N ins	intron	AT	del/del	del/del	del/del	del/del	ins/ins	ins/ins	ins/ins	ins/del
*UGDH*	1N ins	intron	T	del/del	del/del	del/del	del/del	ins/ins	ins/del	ins/ins	ins/del
	1N ins	intron	G	del/del	del/del	del/del	del/del	ins/ins	ins/del	ins/ins	ins/del

N: nucleotide; ins: insertion; del: deletion.

## Discussion

With the rapid development of sequencing technologies and bioinformatics tools, it is now feasible to sequence the complete genome of many species, such as human, mouse and *Arabidopsis* [32–34]. Compared with traditional technologies, NGS has numerous major advantages, including enabling sequencing that is cheaper and can be performed on a larger scale. Moreover, unlike GWASs, it can identify numerous rare and common variants as genetic causes of complex traits, especially in human Mendelian disorders [35, 36]. In this paper, we presented the indels in eight Holstein bulls determined using Illumina sequencing, with the aim of detecting the distribution of variants across the cattle genome and identifying the candidate genes for milk protein and fat traits. To our knowledge, this is one of the first studies involving whole-genome resequencing for milk protein and fat traits in dairy cattle; based on the indels that were polymorphic between the high and low groups, we identified a few candidate genes potentially associated with milk protein and fat traits.

### Indel discovery

Since the first report of the construction of a human indel map [6], numerous indels have been found in different species. In our research, we identified approximately 365,000 short indels for each bull, which was much more than for the Fleckvieh bull (n = 115,000) [37], but slightly fewer than for the Japanese native cattle Kuchinoshima-Ushi (n = 629,256) [38], Chikso (Korean brindle cattle) (n = 551,363) [24], Korean brown cattle (n = 697,048) [39], Jeju black cattle (n = 702,965) [39], Korean Holstein (n = 631,332) [39] and French dairy breeds (n = 873,372) [25], which was probably due to the sequencing fold coverage and different genetic backgrounds. In addition, in this study, the indels were validated by sequencing at a rate as high as 70%, which was the same as in a previous study (70%) [24]. In the current research, approximately 89% of insertions and 88% of deletions were less than 4 bp, and the number of indels in each chromosome declined with decreasing chromosome length. All of these findings were similar to those in previous studies [24, 37, 38]. The distributions in each chromosome and the lengths of the indels determined here were in line with recent findings in other species, such as chicken, Tibetan macaque, Quarter Horse, and sorghum [7–9, 11, 40].

### Candidate genes for milk composition

In the present study, we performed integrated analysis of previously reported QTL, significant SNPs identified by previous GWASs, and the biological functions of genes, which enabled us to identify 11 candidate genes potentially associated with milk protein and fat traits. These genes contained at least one indel that was polymorphic between the high and low groups as described below.

#### Candidate genes containing exonic indel

Indels in a gene coding region, especially frameshift indels, had a major influence on gene function [41, 42]. Among the 11 candidate genes, the *FCGR2B* gene with one frameshift indel in exon 7 encodes a transmembrane glycoprotein that is a member of the low-affinity immunoglobulin gamma Fc receptors and plays a key role in phagocytosis and the clearing of immune complexes. Bovine mammary gland, which is a product of the innate immune system, is active during lactation. *FCGR2B* might affect the milk protein and fat traits through playing an indispensable role in preventing the individuals from disease infection during lactation. *CENPE* with two exonic indels (exons 58 and 68) and seven intronic indels was a component of the kinetochore complex and part of the spindle assembly checkpoint those were responsible for maintain chromosome congression and mitotic checkpoint [43]. It was a candidate gene for triple-negative breast cancer, and played a crucial role in suppressing tumorigenesis [44] Depletion of *CENPE* resulted in promoting the Drosophila epithelial tissues overgrowth [45].

#### Candidate genes with indel in regulatory regions

*RETSAT* also known as *PPSIG* with one indel in 3’ UTR and one indel in intron, was found to be a target gene of PPARα which played an essential role in lipid metabolism and energy balance [46], and responsible for adipogenesis promotion and lipid deposition [47]. *ACSBG2* with one indel in upstream region and one indel in intron encodes a protein that belongs to the acyl-CoA synthetase family, which plays a pivotal role in lipid metabolism and lipid droplet formation. Claire et al. identified *ACSBG2* as a candidate gene that was significantly related to abdominal fat deposition in chicken [48]. In addition, Sun et al. demonstrated that one SNP located in an intron of *ACSBG2* was also significantly associated with yolk development in chicken [49]. *NFKB2* with one indel in upstream region encodes a protein subunit of the transcription factor complex nuclear factor-kappa-B (NFkB), which regulates the transcription of genes related to cell differentiation and proliferation. It was indispensable for normal development of the bovine mammary gland [50]. In our previous study, we identified that a SNP (ARS-BFGL-NGS-107403) close to *NFKB2* gene was significant associated with C14:1 and C14 indices of milk fatty acids in Chinese Holstein population, and the indel identified in current study was only 0.27 Mb away from the significant SNP mentioned above [51].

#### Candidate genes with intronic indel

*TBC1D1* with an intronic indel is a Rab GTPase activating protein, which was involved in AMPK signaling pathway and was important to maintain the glucose and energy homeostasis [52]. Dysfunction of *TBC1D1* in mice induced that lipogeneic gene expressions were up-regulated through IGF1R–PKB–mTOR signaling pathway, including *FASN*, *ACL*, *ACC1* and *SCD* [53]. Polymorphism of *TBC1D1* was found linked to the severe obesity in human [54]. *NLK* with four intronic indels is a serine/threonine-protein kinase, belonging to the MAP kinase (MAPK) subfamily, and is involved in MAPK signaling pathway. Yuan et al. identified the NLK as a negative regulator of mTORC1 complex which was important to the milk protein synthesis [55]. Polymorphisms of *NLK* were strongly associated with obesity in human and fatty acid composition in pig [56, 57].

*MAP3K1* also named as *MEKK1* with one intronic indel, encodes a serine/threonine kinase and is involved in MAPK signaling pathway. Also, it was demonstrated that *MAP3K1* was related to human breast cancer progression [58, 59], thus, *MAP3K1* may play a vital role in keeping bovine mammary gland function well. *SLC30A2* was also known as the zinc transporter ZnT2 with one intronic indel, and played an important role in transporting of Zn into mammary epithelial cells and secreting into the milk [60]. It was found that loss of *ZnT2* can impair mammary gland development and reduce milk composition in mouse [61, 62], and polymorphisms of *ZnT2* were associated with human mammary gland dysfunction [63, 64].

*ANGPT1* also named as *Ang1* with one indel in intron, was one of the ligands for TEK Receptor Tyrosine Kinase (*TEK*) and involved in the PI3K-Akt signaling pathway that is correlated to milk protein synthesis [55]. Mice deficient with *ANGPT1* were shown poor vascular network [65]. Bovine mammary gland was with an advanced microvasculature which were requisite for mammary microcirculation of nutrients transport and supply to maintain milk synthesis and secretory [66]. *UGDH* with two intronic indels also located within a QTL region on chromosome 6 linked to milk protein and fat traits, and its biological function and genetic effects revealed that it was a key candidate gene associated with such traits [67].

Although we identified 11 candidate genes based on the indels in genic and gene flanking regions that were polymorphic between high and low groups, further association analysis should be performed to validate functionally important indels and to characterize more important molecular markers of milk protein and fat traits. Along with the development of the human ENCODE project (2011), similar analyses could be performed for the intergenic variants. This would make it possible to identify more important potential functional variants within the intergenic regions of the genome that play major roles in regulating genes affecting bovine milk composition traits. Therefore, the complete analysis based on a large number of indels in our study should constitute a valuable resource for improving the molecular breeding of dairy cattle.

## Conclusion

In this study, we resequenced eight Holstein bulls and detected 912,302 indels through cattle genome. We also successfully identified 3,625 indels that were polymorphic between high and low groups throughout the genome. Based on the integrated analysis of the differential indels located in genic and flanking regions, previously reported QTL, and significant SNPs identified by GWASs, along with well-known pathways and biological functions of genes, we identified 11 promising candidate genes that may affect milk protein and fat traits of dairy cattle, including *FCGR2B*, *CENPE*, *RETSAT*, *ACSBG2*, *NFKB2*, *TBC1D1*, *NLK*, *MAP3K1*, *SLC30A2*, *ANGPT1* and *UGDH*. And the indels of 11 genes identified in this study will provide a useful resource for future association studies to discovery the major alleles affecting milk composition traits in dairy cattle.

## Supporting Information

S1 File This file contains the detailed information of QTL, significant SNPs and well-known pathways of 11 candidate genes. **Table A.** Detailed information on the reported QTL containing the 8 genes for the high and low groups regarding EBV for milk protein and fat traits. **Table B.** Detailed information of the nearest and most significant SNPs from previous GWASs and well-known pathways to the 11 genes between the high and low groups for milk protein and fat traits.
(DOCX)

S1 Table Summary of validated indels. (XLSX)

S2 Table Functional enrichment of 695 genes containing the indels that were polymorphic between the high and low groups by KOBAS tool. (XLSX)
