# Supplementary material for: Whole-Genome Resequencing of Holstein Bulls for Indel Discovery and Identification of Genes Associated with Milk Composition Traits in Dairy Cattle
Source: PLoS One. 2016 Dec 28;11(12):e0168946. doi: 10.1371/journal.pone.0168946 (PMC5193355; doi:10.1371/journal.pone.0168946)
Supplement: S1 File — Table A. Detailed information on the reported QTL containing the 8 genes for the high and low groups regarding EBV for milk protein and fat traits. Table B. Detailed information of the nearest and most significant SNPs from previous GWASs and well-known pathways to the 11 genes between the high and low groups for milk protein and fat traits. (DOCX) [file pone.0168946.s001.docx]

| **Table A .Detailed information on the reported QTLs containing the 8 genes for the high and low groups regarding EBV for milk protein and milk fat traits.** | | | | | | |
| --- | --- | --- | --- | --- | --- | --- |
| **Gene name** | **Position(bp)** | **Position(cM)** | **Previously reported QTL** | | | |
|  |  |  | **Distance to QTL peak(cM)^1^** | **CI and peak location(cM)** | **Trait** | **References** |
| *FCGR2B* | 7928103-7944645 | chr3:13.30 | 14.17 | 6-32(peak:27.41) | PP | Boichard et al., *Genetics, selection, evolution : GSE, 2003* [1] |
| *CENPE* | 22959282-23042952 | chr6:20.5 | 5.2 | 17.00-34.45(peak:25.7) | PY | Ashwell et al., *J Dairy Sci*, 2004 [2] |
|  |  |  | 8.5 | 23-34(peak:29) | LACTYD | Silva et al., *Genetics and molecular research: GMR*, 2011 [3] |
| *ACSBG2* | 19450258-19499748 | chr7:21.98 | 4.18 | 16.75-39.33(peak:17.8 ) | PP | Ron et al., *J Dairy Sci, 2004* [4] |
| *NFKB2* | 22890250-22898466 | chr26:35.25 | 0.15 | 10.7-64.7(peak:35.4) | DDF | Gautier et al., *Genetics, 2006* [5] |
|  |  |  | 3.55 | 22.862-41.64(peak:31.7) | PY | Plante et al., *J Dairy Sci, 2001* [6] |
| *TBC1D1* | 58838079-59065295 | chr6:65.90 | 1.5 | 35.4-90.1(peak:67.4) | FY FP PY | Chen et al., *J Dairy Sci, 2006* [7] |
|  |  |  | 2.3 | peak:68.2 | DDPY | Rodriguez-Zas et al., *J Dairy Sci*, 2002 [8] |
|  |  |  | 2.3 | 55.2-71(peak:68.2) | DDPY | Freyer et al., *J Anim Breed Genet*, 2002 [9] |
|  |  |  | 2.3 | 57.56-72.43(peak:63.6) | FY | Szyda et al., *J Dairy Sci*,2005 [10] |
|  |  |  | 3.1 | 57.56-72.43(peak:69) | FY PY | Szyda et al., *J Dairy Sci*, 2005 [10] |
|  |  |  | 4.1 | 35.39-97.72(peak:70) | PY PP | Kucerova et al., *J Dairy Sci*, 2006 [11] |
|  |  |  | 4.5 | 53.72-70.74(peak:61.4) | PP | Nadesalingam et al., *Mammalian genome*, 2001 [12] |
| *MAP3K1* | 22358697-22435963 | chr20:27.50 | 4.35 | 20.16-43.54(peak:31.85) | FP PP | Arranz et al., *Animal Genetics*, 1998 [13] |
|  |  |  | 4.36 | 21-50(peak:31.86) | PP | Boichard et al., *Genetics, selection, evolution : GSE*, 2003 [1] |
| *SLC30A2* | 127637570-127645792 | chr2:119.98 | 0.02 | 115.43-130(peak:120) | FY | Harder et al., *J Anim Breed Genet,* 2006 [14] |
| *UGDH* | 60231007-60265969 | chr6:67.46 | 0.06 | 35.4-90.1(peak:67.4) | FY FP | Chen et al., *J Dairy Sci, 2006* [7] |
|  |  |  | 0.06 | 40.4-98(peak:67.4) | PY | Chen et al., *J Dairy Sci, 2006* [7] |
|  |  |  | 0.74 | 55.2-71(peak:68.2) | DDPY | Freyer et al., *J Anim Breed Genet*, 2002 [9] |
|  |  |  | 1.54 | 57.56-72.43(peak:69) | FY PY | Szyda et al., *J Dairy Sci,*2005 [10] |
|  |  |  | 2.54 | 35.39-97.72(peak:70) | PP PY | Kucerova et al., *J Dairy Sci,*2006 [11] |
|  |  |  | 3.86 | 57.56-72.43(peak:63.6) | FY | Szyda et al., *J Dairy Sci,*2005 [10] |
| ^1^The linkage position was estimated relative to UMD3.1.69 and based on the QTL mapper v.2.019 at www.animalgenome.org/cgi-bin/QTLdb/.  PP: milk protein percentage; PY: milk protein yield; LACTYD: milk lactose yield; DDF: milk fat yield (daughter deviation); FY: milk fat yield; DDPY: milk protein yield (daughter deviation). | | | | | | |

| **Table B. Detailed information of the nearest and most significant SNPs from previous GWASs and well-known pathway to the 11 genes between the high and low groups with EBV for milk protein and fat traits.** | | | | | | | | |
| --- | --- | --- | --- | --- | --- | --- | --- | --- |
| **Gene name** | **Gene position (bp)^1^** | **Nearest and most significant SNPs of GWAS** | | | **Traits** | **Raw *p* value** | **References** | **well-known pathway** |
|  |  |  |  |  |  |  |  |  |
|  |  | **Distance (Mb)** | **Name** | **Position (bp)^2^** |  |  |  |  |
|  |  |  |  |  |  |  |  |  |
| *FCGR2B* | 7928103-7944645 | 1.03 | Hapmap54926-ss46526189 | 6900453 | FY PY FP PP | 4.19E-18 1.25E-13 3.66E-17 1.45E-20 | Cole et al., *BMC Genomics, 2011* [15] |  |
|  |  | 1.52 | BFGL-NGS-113990 | 9461404 | FY PY FP PP | 6.43E-29 2.20E-21 1.06E-18 3.31E-19 | Cole et al., *BMC Genomics, 2011* [15] |  |
|  |  | 1.63 | INRA-304 | 9579325 | FY PY FP PP | 7.64E-29 1.86E-21 1.69E-18 3.16E-19 | Cole et al., *BMC Genomics, 2011* [15] |  |
| *CENPE* | 22959282-23042952 | 6.04 | BTA-63194-no-rs | 16922089 | PP | 5.02E-13 | Cole et al., *BMC Genomics, 2011* [15] |  |
|  |  | 6.38 | BFGL-NGS-110788 | 16575847 | FY PY FP PP | 9.01E-10 6.53E-09 7.07E-09 1.71E-14 | Cole et al., *BMC Genomics, 2011* [15] |  |
|  |  | 7.00 | ARS-BFGL-NGS-7249 | 30050205 | PY | 8.38E-08 | Jiang et al., *PLoS ONE*, 2010 [16] |  |
|  |  | 7.26 | Hapmap58425-rs29021238 | 15700204 | FY PY PP | 3.23E-11 1.12E-11 1.08E-08 | Cole et al., *BMC Genomics, 2011* [15] |  |
|  |  | 8.36 | BFGL-NGS-117043 | 14597357 | FY PY FP PP | 4.78E-19 1.40E-14 7.34E-12 3.70E-11 | Cole et al., *BMC Genomics, 2011* [15] |  |
| *RETSAT* | 49475071-49489800 | 0.26 | ARS-BFGL-NGS-75350 | 49734417 | PP | 5.02E-10 | Cole et al., *BMC Genomics, 2011* [15] |  |
|  |  | 0.75 | Hapmap43460-BTA-111292 | 50241607 | FY PY | 1.70E-14 8.75E-10 | Cole et al., *BMC Genomics, 2011* [15] |  |
|  |  | 0.77 | BTB-01660145 | 50264058 | FY PY | 2.00E-11 1.07E-07 | Cole et al., *BMC Genomics, 2011* [15] |  |
|  |  | 1.67 | ARS-BFGL-NGS-1792 | 47803744 | FY PY | 1.17E-15 2.45E-12 | Cole et al., *BMC Genomics, 2011* [15] |  |
|  |  | 2.47 | Hapmap49363-BTA-89255 | 51963535 | FY | 6.95E-09 | Cole et al., *BMC Genomics, 2011* [15] |  |
|  |  | 2.71 | ARS-BFGL-BAC-11717 | 52195257 | FY PY FP | 1.47E-12 1.65E-07 3.38E-09 | Cole et al., *BMC Genomics, 2011* [15] |  |
|  |  | 4.91 | ARS-BFGL-NGS-47330 | 44562022 | FY PY FP PP | 2.48E-08 1.57E-09 3.39E-09 4.40E-20 | Cole et al., *BMC Genomics, 2011* [15] |  |
| *ACSBG2* | 19450258-19499748 | 3.05 | Hapmap23865-BTA-111820 | 16397706 | PP | 1.51E-08 | Cole et al., *BMC Genomics, 2011* [15] | PPAR |
| *NFKB2* | 22890250-22898466 | 0.15 | ARS-BFGL-NGS-9920 | 23048759 | FY PY FP PP | 2.32E-18 1.98E-16 6.07E-16 3.17E-25 | Cole et al., *BMC Genomics, 2011* [15] | MAPK |
|  |  | 1.09 | ARS-BFGL-NGS-7203 | 21800887 | FY PY FP PP | 1.23E-25 1.18E-20 5.98E-12 3.58E-11 | Cole et al., *BMC Genomics, 2011* [15] |  |
|  |  | 2.82 | BFGL-NGS-115980 | 20065602 | FY PY FP PP | 5.15E-09 2.01E-10 4.76E-12 1.10E-28 | Cole et al., *BMC Genomics, 2011* [15] |  |
|  |  | 2.88 | ARS-BFGL-NGS-10103 | 20012464 | FY PY FP PP | 4.41E-12 3.62E-16 3.43E-09 1.09E-25 | Cole et al., *BMC Genomics, 2011* [15] |  |
|  |  | 3.28 | Hapmap51475-BTA-62174 | 19605912 | FY PY FP PP | 6.47E-13 2.42E-16 3.14E-09 3.33E-24 | Cole et al., *BMC Genomics, 2011* [15] |  |
|  |  | 4.08 | BFGL-NGS-114539 | 18808408 | FY | 1.35E-07 | Cole et al., *BMC Genomics, 2011* [15] |  |
|  |  | 4.21 | BFGL-NGS-111739 | 18684200 | FY PY | 5.52E-12 1.50E-09 | Cole et al., *BMC Genomics, 2011* [15] |  |
| *TBC1D1* | 58838079-59065295 | 2.13 | ARS-BFGL-NGS-106015 | 61199572 | FY PY | 1.09E-15 7.16E-16 | Cole et al., *BMC Genomics, 2011* [15] | AMPK |
|  |  | 2.43 | Hapmap39208-BTA-68393 | 61492196 | FY PY FP PP | 9.42E-22 3.71E-18 2.10E-20 8.81E-30 | Cole et al., *BMC Genomics, 2011* [15] |  |
| *NLK* | 20177847-20307088 | 0.09 | ARS-BFGL-NGS-106227 | 20089776 | PP | 5.59E-08 | Cole et al., *BMC Genomics, 2011* [15] | MAPK |
| *MAP3K1* | 22358697-22435963 | 2.77 | ARS-BFGL-BAC-33654 | 19587484 | FY PY FP PP | 2.28E-12 8.54E-16 8.83E-08 1.61E-20 | Cole et al., *BMC Genomics, 2011* [15] | MAPK |
|  |  | 2.84 | Hapmap50711-BTA-49985 | 19520788 | FY PY FP PP | 1.49E-14 2.36E-14 6.39E-10 4.35E-17 | Cole et al., *BMC Genomics, 2011* [15] |  |
|  |  | 3.17 | BTB-00775883 | 19187276 | FY PY PP | 2.56E-12 7.26E-16 1.86E-07 | Cole et al., *BMC Genomics, 2011* [15] |  |
|  |  | 3.29 | BTB-00775794 | 19067657 | FY PY | 1.30E-08 5.12E-12 | Cole et al., *BMC Genomics, 2011* [15] |  |
| *SLC30A2* | 127637570-127645792 | 0.79 | BTA-31250-no-rs | 128437731 | PY FP PP | 3.25E-08 3.75E-10 1.26E-24 | Cole et al., *BMC Genomics, 2011* [15] |  |
|  |  | 3.92 | ARS-BFGL-NGS-69013 | 123717269 | FY PY FP PP | 5.36E-22 2.61E-16 1.46E-11 3.26E-09 | Cole et al., *BMC Genomics, 2011* [15] |  |
| *ANGPT1* | 59136610-59436693 | 1.55 | Hapmap24398-BTC-062971 | 57584280 | PP | 8.28E-09 | Cole et al., *BMC Genomics, 2011* [15] | PI3K-Akt |
|  |  | 2.73 | ARS-BFGL-NGS-29452 | 62166389 | PP | 1.04E-09 | Cole et al., *BMC Genomics, 2011* [15] |  |
| *UGDH* | 60231007-60265969 | 0.93 | ARS-BFGL-NGS-106015 | 61199572 | FY PY | 1.09E-15 7.16E-16 | Cole et al., *BMC Genomics, 2011* [15] |  |
|  |  | 1.23 | Hapmap39208-BTA-68393 | 61492196 | FY PY FP PP | 9.42E-22 3.71E-18 2.1E-20 8.81E-30 | Cole et al., *BMC Genomics, 2011* [15] |  |
| ^1,2^Means the position on the bovine genome sequence of UMD3.1.69. FY: milk fat yield; PY: milk protein yield; FP: milk fat percentage; PP: milk protein percentage. | | | | | | | | |

**Reference**

1. Boichard D, Grohs C, Bourgeois F, Cerqueira F, Faugeras R, Neau A, et al. Detection of genes influencing economic traits in three French dairy cattle breeds. Genetics Selection Evolution. 2003;35(1):77-102.

2. Ashwell M, Heyen D, Sonstegard T, Van Tassell C, Da Y, VanRaden P, et al. Detection of quantitative trait loci affecting milk production, health, and reproductive traits in Holstein cattle. Journal of dairy science. 2004;87(2):468-75.

3. Silva A, Azevedo A, Gasparini K, Verneque R, Peixoto M, Panetto B, et al. Quantitative trait loci affecting lactose and total solids on chromosome 6 in Brazilian Gir dairy cattle. Genetics and Molecular Research. 2011;10(4):3817-27.

4. Ron M, Feldmesser E, Golik M, Tager-Cohen I, Kliger D, Reiss V, et al. A complete genome scan of the Israeli Holstein population for quantitative trait loci by a daughter design. Journal of dairy science. 2004;87(2):476-90.

5. Gautier M, Barcelona RR, Fritz S, Grohs C, Druet T, Boichard D, et al. Fine mapping and physical characterization of two linked quantitative trait loci affecting milk fat yield in dairy cattle on BTA26. Genetics. 2006;172(1):425-36.

6. Plante Y, Gibson J, Nadesalingam J, Mehrabani-Yeganeh H, Lefebvre S, Vandervoort G, et al. Detection of quantitative trait loci affecting milk production traits on 10 chromosomes in Holstein cattle. Journal of dairy science. 2001;84(6):1516-24.

7. Chen H, Zhang Q, Yin C, Wang C, Gong W, Mei G. Detection of quantitative trait loci affecting milk production traits on bovine chromosome 6 in a Chinese Holstein population by the daughter design. Journal of dairy science. 2006;89(2):782-90.

8. Rodriguez-Zas SL, Southey BR, Heyen DW, Lewin HA. Interval and composite interval mapping of somatic cell score, yield, and components of milk in dairy cattle. J Dairy Sci. 2002;85(11):3081-91. doi: 10.3168/jds.S0022-0302(02)74395-6. PubMed PMID: 12487475.

9. Freyer G, Kühn C, Weikard R, Zhang Q, Mayer M, Hoeschele I. Multiple QTL on chromosome six in dairy cattle affecting yield and content traits. Journal of Animal Breeding and Genetics. 2002;119(2):69-82.

10. Szyda J, Liu Z, Reinhardt F, Reents R. Estimation of quantitative trait loci parameters for milk production traits in German Holstein dairy cattle population. Journal of dairy science. 2005;88(1):356-67.

11. Kučerová J, Lund MS, Sørensen P, Sahana G, Guldbrandtsen B, Nielsen VH, et al. Multitrait quantitative trait loci mapping for milk production traits in Danish Holstein cattle. Journal of dairy science. 2006;89(6):2245-56.

12. Nadesalingam J, Plante Y, Gibson JP. Detection of QTL for milk production on Chromosomes 1 and 6 of Holstein cattle. Mammalian Genome. 2001;12(1):27-31.

13. Arranz JJ, Coppieters W, Berzi P, Cambisano N, Grisart B, Karim L, et al. A QTL affecting milk yield and composition maps to bovine chromosome 20: a confirmation. Animal genetics. 1998;29(2):107-15.

14. Harder B, Bennewitz J, Reinsch N, Thaller G, Thomsen H, Kühn C, et al. Mapping of quantitative trait loci for lactation persistency traits in German Holstein dairy cattle. Journal of Animal Breeding and Genetics. 2006;123(2):89-96.

15. Cole JB, Wiggans GR, Ma L, Sonstegard TS, Lawlor TJ, Jr., Crooker BA, et al. Genome-wide association analysis of thirty one production, health, reproduction and body conformation traits in contemporary U.S. Holstein cows. BMC genomics. 2011;12:408. doi: 10.1186/1471-2164-12-408. PubMed PMID: 21831322; PubMed Central PMCID: PMC3176260.

16. Jiang L, Liu J, Sun D, Ma P, Ding X, Yu Y, et al. Genome wide association studies for milk production traits in Chinese Holstein population. PloS one. 2010;5(10):e13661.
